# 129. Antimicrobial Resistance Genes Were Reduced Following Administration of Investigational Microbiota-Based Live Biotherapeutic RBX2660 to Individuals with Recurrent *Clostridioides difficile* Infection

**DOI:** 10.1093/ofid/ofab466.129

**Published:** 2021-12-04

**Authors:** Heidi Hau, Dana M Walsh, Carlos Gonzalez, Bill Shannon, Ken Blount

**Affiliations:** 1 Rebiotix Inc, Roseville, Minnesota; 2 Rebiotix, Inc., Roseville, MN; 3 BioRankings, LLC, St. Louis, Missouri

## Abstract

**Background:**

Intestinal colonization by antimicrobial resistant (AMR) pathogens is a known health and infection risk, and is common among individuals with recurrent *Clostridioides difficile* infections (rCDI). Accordingly, therapeutic approaches that decolonize the gut of AMR pathogens could be valuable to patients to reduce risk of associated illnesses. Herein, we assessed gut colonization with AMR bacteria before and after treatment with RBX2660—a microbiota-based investigational live biotherapeutic—in the PUNCH CD3 Phase 3 trial for reducing CDI recurrence.

**Methods:**

rCDI participants enrolled in PUNCH CD3 received a blinded single dose of RBX2660 or placebo within 24 to 72 hours after completing antibiotic treatment for the most recent rCDI episode. Clinical response was the absence of CDI recurrence at eight weeks after treatment, and participants were asked to submit stool samples prior to RBX2660 or placebo treatment (baseline) and 1, 4 and 8 weeks, 3 and 6 months after study treatment. Samples were extracted and sequenced using a shallow shotgun method. The presence and number of AMR genes was determined for 175 participant samples and 116 RBX2660 samples using 90% K-mer sequence coverage based on the MEGARes database. AMR gene count data were collapsed into count columns to adjust for sparseness in the matrices and were analyzed using a Generalized Linear Mixed Model.

**Results:**

Clinically, RBX2660 demonstrated superior efficacy versus placebo (70.4% and 58.1%, respectively), and the total AMR genes per participant decreased significantly from before to after treatment in RBX2660-treated responders (p< .05, Figure 1) and remained low to at least 6 months. Among genes that decreased in RBX2660 responders were clinically important extended-spectrum beta-lactamase (*bla*_TEM_, *bla*_SHV_, *bla*_CTX-M_), vancomycin resistance (*vanA*, *vanB*), and fluoroquinolone resistance genes (*gyrA*, *parC*).

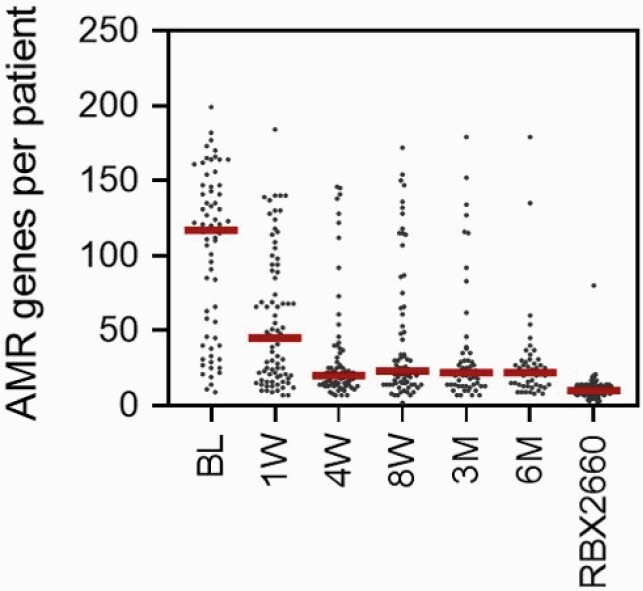

Total AMR genes per PUNCH CD3 participant among RBX2660-treated responders at the indicated time points and in the RBX2660 investigational product. The red lines indicate timepoint group medians.

**Conclusion:**

In the PUNCH CD3 Phase 3 trial of RBX2660 for rCDI, AMR gene content decreased after RBX2660 treatment and remained low to at least 6 months, consistent with prior RBX2660 trials. This underscores the potential of microbiota-based biotherapeutics for decolonizing AMR bacteria from gut microbiota and thereby reducing AMR infection risks.

**Disclosures:**

**Heidi Hau, PhD**, **Rebiotix Inc.** (Employee) **Dana M. Walsh, PhD**, **Rebiotix** (Employee) **Ken Blount, PhD**, **Rebiotix Inc., a Ferring Company** (Employee)

